# Recombinant bacteriophage T4 Rnl1 impacts Streptococcus mutans biofilm formation

**DOI:** 10.1080/20002297.2020.1860398

**Published:** 2020-12-24

**Authors:** Juxiu Chen, Zhanyi Chen, Keyong Yuan, Zhengwei Huang, Mengying Mao

**Affiliations:** a Department of Endodontics, Shanghai Ninth People’s Hospital, College of Stomatology, Shanghai Jiao Tong University School of Medicine, Shanghai, China; b National Clinical Research Center for Oral Diseases, Shanghai, China; c Shanghai Key Laboratory of Stomatology & Shanghai Research Institute of Stomatology, Shanghai, China

**Keywords:** Biofilm, T4 Rnl1, exopolysaccharides, *Streptococcus mutans*, dental caries

## Abstract

Bacteriophage T4 RNA ligase 1 (T4 Rnl1) can be stably expressed in many bacteria and has been reported to affect the bioactivity of the host bacteria. Recently, we constructed bacteriophage T4 Rnl1 expressing system in Streptococcus mutans, a crucial biofilm-forming and dental caries-causing oral pathogen. Here, we characterized the function of recombinant bacteriophage T4 Rnl1 in biofilm formation of S. mutans. The T4 Rnl1 mutant exhibited similar growth phenotype but resulted in a significant reduction of biofilm biomass compared to wild type strain and empty plasmid carrying strain. The abnormal biofilm of the T4 Rnl1 mutant harbored loose bacterial clusters with defective production and distribution of exopolysaccharides. Moreover, the expression of several biofilm formation-associated genes was dysregulated at mRNA level in the T4 Rnl1 mutant. These results reveal that the bacteriophage T4 Rnl1 exert antibiofilm activities against the cariogenic bacterium S. mutans, which impacts the spatial organization of the exopolysaccharides and further impairs the three-dimensional biofilm architecture. These findings implicate that manipulation of bacteriophage T4 Rnl1, a biological tool used for RNA ligation, will provide a promising approach to cariogenic biofilm control.

## Introduction

The ecology of the oral microbiome significantly affects the oral health status. Beyond bacteria, bacteriophages are thought to play a critical role in shaping the oral microbiome [1]. Recently, the increasing attention has been given to the phage therapy. Phages can be used to attack the target pathogens and prevent oral infectious disease development, such as the most prevalent, dental caries [2]. It has been reported that a new phage, ɸAPCM01, can infect *Streptococcus mutans* DPC6143, one of the principal agents of dental caries formation, further impacting on its growth and biofilm formation [3]. Bacteriophages are highly evolved nanomachines that recognize bacterial cell walls to deliver genetic information [4]. However, the exquisite specificity against pathogens limits phage therapy to common use [5]. Bacteriophage T4 RNA ligase 1 (T4 Rnl1) is a tRNA repair enzyme that functions in phage infection and has been found to be stably expressed and work in many bacteria via recombinant vector [6–8]. The expression of bacteriophage virulent proteins like T4 Rnl1 in target bacteria by a recombinant vector might provide an alternative strategy to overcome the narrow spectrum of the traditional phage therapy. As the founding member of the RNA ligase family, T4 Rnl1 can facilitate a preferential ligation of the 3ʹ and 5ʹ ends of RNA and has been used as a biological tool used for identifying direct targets of bacterial small regulatory RNA in many bacteria [9,10]. The expressed recombinant T4 Rnl1 could negatively affect cell viability in *Pseudomonas aeruginosa* [6,8]. Recently, we firstly established the T4 Rnl1 expression system in *S. mutans* for detecting the targets of sRNAs [7]. However, the biofunctional effect of recombinant T4 Rnl1 on *S. mutans* remains unclear.

*S. mutans* is generally regarded as a primary dental caries-causing oral pathogen, and also a typical biofilm-forming bacterium [11]. Dental caries is one of the most prevailing and persistent diseases in the human population [12], and cariogenic biofilm is a prerequisite to dental caries. Biofilm provides protection to pathogens against environmental inimical influences and thus improves the virulence of pathogenic bacteria [13,14]. *S. mutans* contributes greatly to the formation of the cariogenic biofilm. The *gtf* gene family, known as the encoding model of GtfB, -C, and – D in *S. mutans*, can synthesize exopolysaccharide from sucrose. Briefly, GtfB, which synthesizes mostly insoluble glucan (α1,3-linked); GtfD, which synthesizes soluble glucan (α1,6-linked); and GtfC, which synthesizes a mixture of insoluble and soluble glucans [15]. In particular, *S. mutans* initially attaches to the tooth surface and contributes to the exopolysaccharides formation of the cariogenic biofilm matrix, thereby accelerating the maturation of cariogenic biofilm [16,17]. Hence, investigations to regulate *S. mutans* biofilm formation have continued to dominate the caries management field for decades.

In this study, we aimed to investigate what effect of bacteriophage T4 Rnl1 has on the cariogenic *S. mutans*. The present study combines plasmid recombination technology and *in vivo* T4 Rnl1 ligation assay to construct and confirm the T4 Rnl1 mutant of *S. mutans*. Furthermore, microscopy and biofilm assessment were combined to evaluate the effect of T4 Rnl1 on bacterial clustering, exopolysaccharides assembly and biofilm formation in *S. mutans*. We report that the recombinant bacteriophage T4 Rnl1 can impact on the spatial organization of the exopolysaccharides and further impair the biofilm formation of *S. mutans*. Manipulation of bacteriophage T4 Rnl1 may thus provide a new approach to control cariogenic biofilm.

## Materials and methods

### Bacterial strains and growth conditions

The bacterial strains and plasmids used in this study are listed in Table 1. *S. mutans* UA159 and its derivatives were grown on brain heart infusion (BHI) media (Oxoid, Basingstoke, UK) at 37°C in a 20% CO_2_ and 80% N_2_ aerobic atmosphere. For the biofilm assay, the medium was supplemented with 1% sucrose (BHIS).

**Table 1.** Bacterial strains and plasmid used in this study

Strain or plasmid	Description	Source or reference
WT	*Streptococcus mutans* UA159	ATCC 700610
WT_B	*S. mutans* UA159 carrying pDL278 vector	This study
WT_T	*S. mutans* UA159 carrying pDL278_*t4rnl1* vector	This study
pDL278	*Escherichia coli-Streptococcus* shuttle vector and expression plasmid (spectinomycin)	Novagen
pDL278_*t4rnl1*	*t4rnl1* gene cloned into pDL278 for expression of T4 Rnl1	This study

### Construction of the recombinant T4 Rnl1 mutant strain

The oligonucleotides used in this study are presented in Table 2. All primers designed using MacVector 7.0 software were purchased commercially (Sangon Biotech, Shanghai, China). *S. mutans* UA159, referred to as the wild type (WT) strain, was used as a recipient for mutant construction. The shuttle vector pDL278 was used to express the *t4rnl1* gene under the control of the *S. mutans* endogenous promoter region of the *vicR* gene as previous study described [7]. The *t4rnl1* and *vicR* promoter sequences were obtained by oligonucleotide synthesis (Sangon Biotech, Shanghai, China). The 1,495-bp amplicons were cloned into the vector pDL278 at Smal and SalI restriction sites generating recombinant plasmid pDL278_*t4rnl1*. The pDL278 blank and the pDL278_*t4rnl1* plasmids were, respectively, transformed into WT strains. The resulting *t4rnl1* expressed WT strain was named WT_T, while the one carrying pDL278 blank vector was named WT_B. Mutant strains were isolated on BHI-spec agar plates (spectinomycin, 1 mg/mL) and were further verified by colony PCR.

**Table 2.** List of oligonucleotide primers used in this study

Primers	Nucleotide sequences	Relevant purpose
**Cloning^a^**		
***t4rnl1*+ pro-F**	5ʹ GGTA**CCCGGG**GGATCCAGTCTTC 3’	Construction of pDL278_*t4rnl1*
***t4rnl1*+ pro-R**	5ʹ GCAGGTCGACTTAGTATCCTTCTGG 3’	
**Ligation assay**		
**tRNA_Gly-F**	5ʹ CTACAGCCTTCCAAGCTGTTGTC 3’	Confirmation of T4 Rnl1 ability *in vivo*
**tRNA_Ser-R**	5ʹ GGGATTCGAACCCACGCACG 3’	
**Primers**	**Nucleotide sequences**	**Amplification size (bp)**
**qRT-PCR**		
***gyrA*-F**	5ʹ ATTGTTGCTCGGGCTCTTCCAG 3’	105
***gyrA*-R**	5ʹ ATGCGGCTTGTCAGGAGTAACC 3’	
***t4rnl1*-F**	5ʹ CTCAGATGATGTAAGTGCATCTGGAAG 3’	109
***t4rnl1*-R**	5ʹ CATAATTCCACGACATTCTAGTGCATC 3’	
***gtfB*-F**	5ʹ ACACTTTCGGGTGGCTTG 3’	127
***gtfB*-R**	5ʹ GCTTAGATGTCACTTCGGTTG 3’	
***gtfC*-F**	5ʹ CCAAAATGGTATTATGGCTGTCG 3’	136
***gtfC*-R**	5ʹ TGAGTCTCTATCAAAGTAACGCAG3’	
***gtfD*-F**	5ʹ AATGAAATTCGCAGCGGACTTGAG 3’	245
***gtfD*-R**	5ʹ TTAGCCTGACGCATGTCTTCATTGTA 3’	
***gbpB*-F**	5ʹ AGCAACAGAAGCACAACCATCAG 3’	150
***gbpB*-R**	5ʹ CCACCATTACCCCAGTAGTTTCC 3’	
***dexA*-F**	5ʹ AGGGCTGACTGCTTCTGGAGT 3’	142
***dexA*-R**	5ʹ AGTGCCAAGACTGACGCTTTG 3’	
***rnc*-F**	5ʹ CAGCCTCTTGCTCTGCTAATTTT 3’	150
***rnc*-R**	5ʹ AAGTTGACGGGGATGTTTTGAT 3’	

a. Smal restriction sites are in boldface, and SalI restriction sites are underlined.

### T4 Rnl1 ligation assay

The ability of T4 Rnl1 *in vivo* was determined by ligation assay with some modifications [6]. The mutant and WT strains grown under planktonic conditions were harvested at mid-exponential phase on BHI and BHI-spec media, respectively. RNA was extracted according to the classic TRIzol-chloroform protocol (Invitrogen, Carlsbad, CA, USA). Contaminating genomic DNA was removed by Turbo RNase-free DNase I (Ambion, Austin, TX, USA) according to the manufacturer’s instructions. The purity (A260/A280) and concentration of RNA were determined using a NanoDrop2000 spectrophotometer (Thermo Scientific, Waltham, MA, USA). PCR and agarose gel electrophoresis were carried out to confirm that the template was free of DNA contamination. RNA was reverse transcribed to cDNA using random hexamers by the RevertAid First Strand cDNA Synthesis Kit (Thermo Scientific, Waltham, MA, USA). PCR amplification used a Premix Taq^TM^ Mix (TaKaRa, Kusatsu, Shiga, Japan). The specific primer pair (tRNA_Gly-F and tRNA_Ser-R), applied to detect tRNA Gly-Ser chimeras, was listed in Table 2. Cycling conditions were: 95°C for 3 min; 30 cycles of 94°C for 25 s, 58°C for 25 s and 72°C for 60 s; and a final cycle of 72°C for 5 min. The PCR products were separated by 2% agarose gel electrophoresis.

### Planktonic growth assay

To determine whether the recombinant T4 Rnl1 had an impact on the growth of *S. mutans*, overnight cultures of WT, WT_B, and WT_T strains were subcultured in BHI until the OD_600 nm_ reached 0.5 and inoculated at a dilution of 1:100 into fresh BHI broth [18]. For the optical density method, the growth of these planktonic bacteria was monitored using a 96‐well cell culture plate (Corning, NY, USA) incubated at 37°C for 24 hr. The optical density of the cell culture was measured hourly at 600 nm by a microplate reader with each individual well covered with sterile mineral oil and maintained at 37°C. Wells with medium only (without bacteria) were used as blanks.

### Biofilm formation assay

A crystal violet (CV) microtiter assay for analyzing biofilm biomass of WT, WT_T and WT_B was performed as previously described [19]. Briefly, overnight cultures of mutant and WT strains were diluted into fresh BHI and grown to the mid-exponential phase. Aliquots of 2 μL cultures of bacteria (OD_600 nm_ = 0.5) and 200 μL pre-warmed BHIS were added to each well of a 96‐well cell culture plate and then incubated anaerobically for 24 hr. Then, the formed biofilms were washed three times with phosphate-buffered saline (pH 7.2) to remove non-adherent cells, air dried, and stained with 0.1% (w/v) CV for 15 min. The stained biofilms were thoroughly washed with distilled water and resuspended in 1 mL destaining solution (ethanol: acetone 8: 2). Biofilm formation was quantified by measuring the optical density of the solution at 575 nm.

### Scanning electron microscopy

To observe the architecture of the mutant and WT *S. mutans* biofilms, scanning electron microscopy (SEM; FEI, Hillsboro, OR, USA) was performed. Aliquots of 0.5 mL mid-exponential cultures (OD_600 nm_ = 0.5) of each strain and 2 mL BHIS were added on round glass slides (diameter = 8 mm) placed on the bottom of wells of a 24-well plate. After anaerobic incubation for 24 hr, biofilms formed on slides were fixed in a bath containing 2.5% glutaraldehyde for 12 h at room temperature. Formed biofilms were consequently rinsed in PBS and dehydrated in sequential ethanol. Samples were then sputter-coated under vacuum with gold and observed at 5,000 x, 10,000 x and 20,000 x magnification. The images are representative of at least three independent observations.

### Confocal laser scanning microscopy

To determine the production and distribution of exopolysaccharides in the mutant and WT *S. mutans* biofilms, confocal laser scanning microscopy (CLSM; TSP SP2; Leica, Germany) based on *in situ* labeling of the exopolysaccharides with 1 μM Alexa Fluor 647 (Invitrogen, Eugene, OR, USA) and bacterial cells with 2.5 μM SYTO 9 (Invitrogen, Carlsbad, CA, USA) were applied. Each sample was observed using Ar (514/488 nm) and He-Ne (543 nm) lasers. Green indicated bacteria and red indicated exopolysaccharides. Three-dimensional images were generated with Imaris 7.0.0 software (Bitplane, Zurich, Switzerland).

### RNA extraction, reverse transcription, and qRT-PCR analysis

For total RNA extraction, mutant and WT *S. mutans* strains were cultured at mid-exponential phase in BHI media. Total RNA was extracted from *S. mutans* cells according to the classic TRIzol-chloroform protocol (Invitrogen, Carlsbad, CA, USA), as templates for the genomic DNA removing with the Turbo RNase-free DNase I (Ambion, Austin, TX, USA). Total RNA reverse transcription was performed with the RevertAid First Strand cDNA Synthesis Kit (Thermo Scientific, Waltham, MA, USA). qRT-PCR was conducted as described by the manufacturer using a Bio-Rad CFX96 TM Real-Time System (Bio-Rad, Hercules, CA, USA) and the QuantiTect SYBR- Green PCR kit (Qiagen, Valencia, CA, USA), with specific primers for *gyrA, gtfB/C/D, gbpB, dexA* and *rnc*. All primers for qRT-PCR were obtained commercially (Sangon Biotech, Shanghai, China) and are listed in Table 2. Threshold cycle values (CT) were quantified and the expression of each gene was normalized relative to the expression of the *gyrA* gene, and according to the 2^−ΔΔCT^ method.

### Statistical analyses

The normal distribution and the homogeneity of variances of data were determined by the Shapiro-Wilk test and the Bartlett test, respectively. The Fisher tests and one-way ANOVA were applied to evaluate the statistical significance of the differences for parametric variables using SPSS 16.0 (SPSS Inc., Chicago, IL, USA), while the Kruskal-Wallis test and least significant difference multiple comparisons were applied for nonparametric variables. Differences between means of data were considered statistically significant if the *p* value was less than 0.05. All experiments were repeated three times with at least three biological replicates.

## Results

### The construction and confirmation of T4 Rnl1 mutant

**Figure 1.**
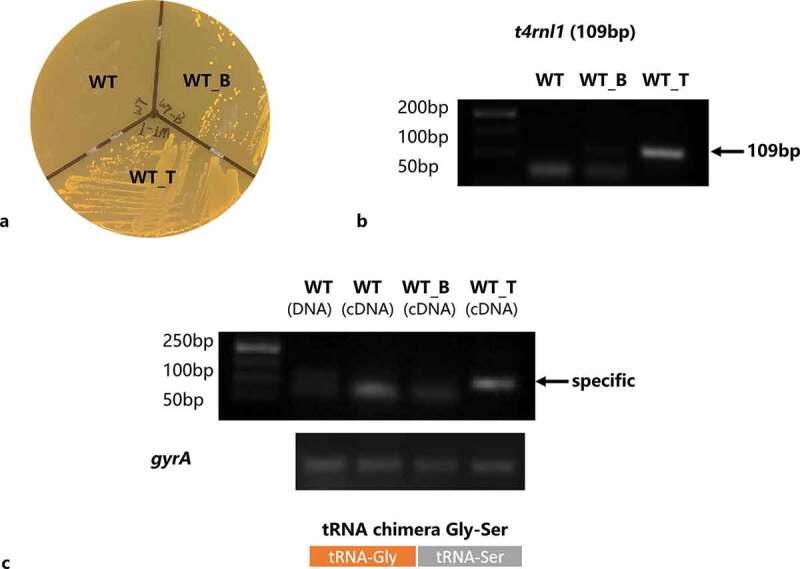
Construction and confirmation of *Streptococcus mutans* T4 Rnl1 mutant. **a** Incubated for 24 hr on a spectinomycin selective plate. Positive colonies were obtained in mutant strains carrying spectinomycin-resistance pDL278 vectors. **b** Colony PCR products of wild type (WT) and mutant (WT_B for WT carrying pDL278 blank vector, WT_T for WT carrying pDL278_*t4rnl1* vector) strains pictured by agarose gel electrophoresis. The WT_T mutant strain showed the specific band around 100 bp (*t4rnl1* fragment is 109 bp), while the other two strains did not. **c** Detection of the ligated transfer RNA chimeras of tRNA-Gly and tRNA-Ser by RT-PCR. The specific band could be seen in the WT_T mutant strain

After 24-h incubation on a spectinomycin selective plate, positive colonies could be seen in the WT_T and WT_B strains area. The WT strain was sensitive to spectinomycin and thus failed to survive (Figure 1a). It was verified that the pDL278 vector had a stable expression in the *S. mutans* strain. Next, colony PCR was applied to evaluate the existence of the *t4rnl1* gene in the WT_T strain with the template of the bacteria colony picked up from the BHI plate. The specific band near ~100 bp could be only be seen in the WT_T strain (Figure 1b). Besides, the ability of *t4rnl*-encoded T4 Rnl1 was checked by *in vivo* ligation assay. After PCR amplification with the forward primer of tRNA_glycine and the reverse primer of tRNA_serine, the presence of specific glycine-serine chimeras, ligated by T4 Rnl1, was detected. As shown in Figure 1c, the specific amplicon was pictured in the WT_T strain alone, not in the other two other strains, which testified the bioactivity of T4 Rnl1. Taken together, the recombinant T4 Rnl1 could stably express and function in *S. mutans*.

### The T4 Rnl1 mutant demonstrates reduced biofilm mass with similar growth phenotype

**Figure 2.**
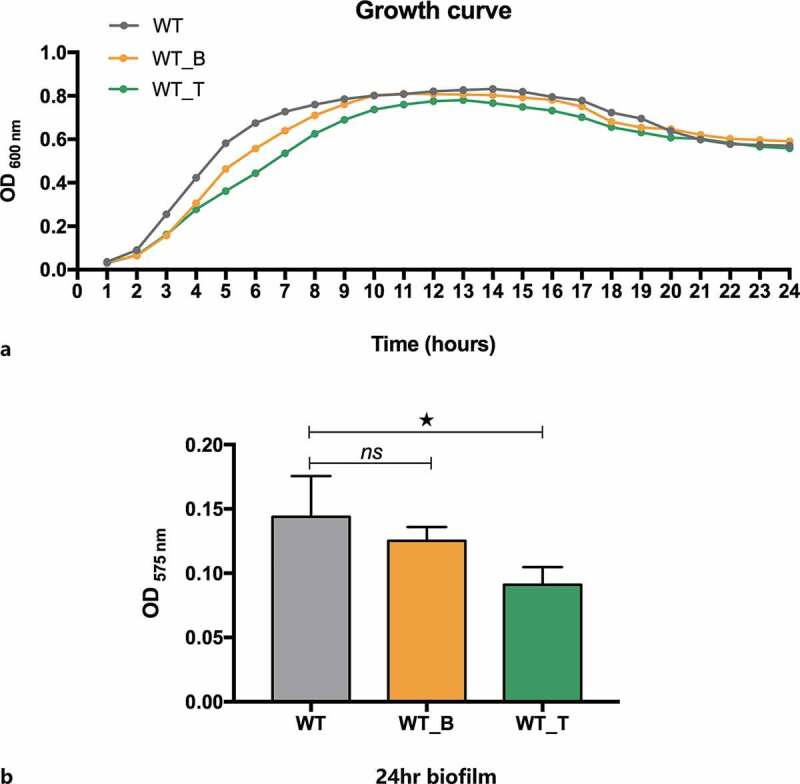
**A** Growth curves of WT and mutant strains in planktonic cultures. Each experiment was repeated 10 times. **b** WT and mutant strains were allowed to form stable biofilms for 24 hr in brain heart infusion (BHI) plus 1% sucrose biofilm medium. Their biomass was quantified by crystal violet staining, and the optical density at 575 nm was read. The data represent 10 biological replicates and are presented as the mean ± standard deviation. * Kruskal-Wallis test *p*< 0.05

To compare the cell growth capacity of the WT and mutant strains in detail, the planktonic growth assay was conducted to picture the 24-h growth curve. While the growth rate of the WT_T mutant in planktonic culture was very similar to that of the WT and WT_B strains (Figure 2a). Slightly, The growth rate of the WT_T strain was slightly lower than that of the other two strains when reaching the peak. Next, the specific ability of the WT, WT_B, and WT_T strains to form stable biofilms was evaluated. The bacteria were cultured in BHIS for 24 hours to form mature biofilms, and the biomass (dry weight) was quantified by CV staining. The biomass of 24-hour *S. mutans* biofilm in the WT_T strain was lower than that of WT, which revealed that the expression of T4 Rnl1 led to a reduction in mature biofilm formation when compared to the WT strain (Figure 2b; *p*< 0.05).

### The T4 Rnl1 mutant biofilm contains smaller microcolonies and displays a defective matrix

**Figure 3.**
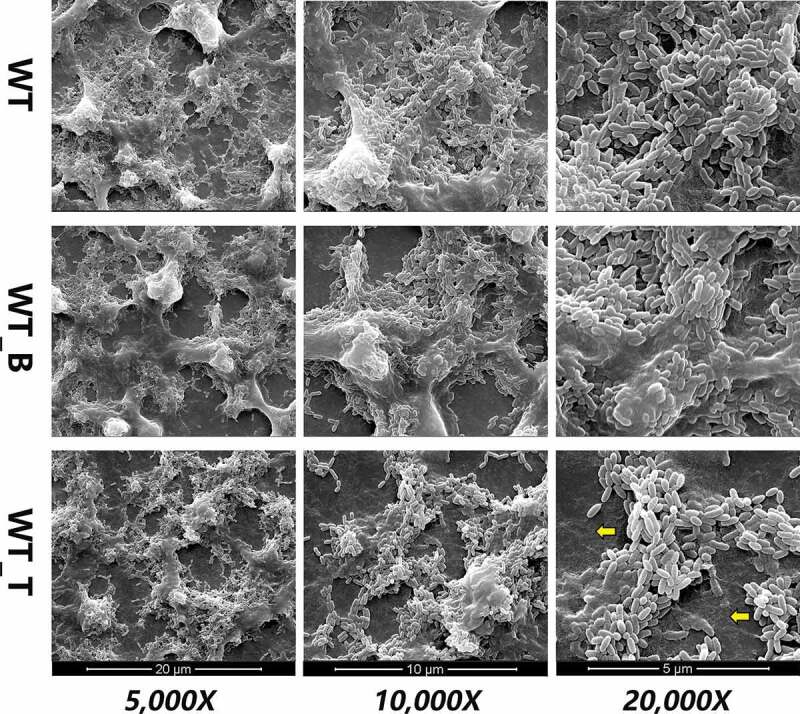
Scanning electron microscopy observations of the architecture of *S. mutans* WT, WT_B and WT_T strain biofilms. WT_T showed decreased extracellular polymeric substances in the biofilms interspersed among ‘blank’ areas (yellow arrows). Representative images are shown from at least six randomly selected positions of each sample

To detect how T4 Rnl1 affected *S. mutans* biofilm formation, the biofilm morphogenesis of WT, WT_B and WT_T strains was first observed by SEM at first (Figure 3). After 24-h incubation, the biofilm of the WT strain was complete and reticular, where bacteria were packed within the enriched extracellular polymeric substances (EPS) and formed into dense microcolonies. In contrast, the biofilm architecture of the WT_T strain was much sparser, presenting a poorly established biofilm structure. Under a higher magnification (20,000 x), more scattered microcolonies with less EPS of the WT_T biofilm could be seen. It was clearly detected that the T4 Rnl1 mutant showed immature biofilm architecture, indicating that T4 Rnl1 is involved in the regulation of biofilm composition and structure.

### The T4 Rnl1 mutant impedes exopolysaccharide production and assembly

**Figure 4.**
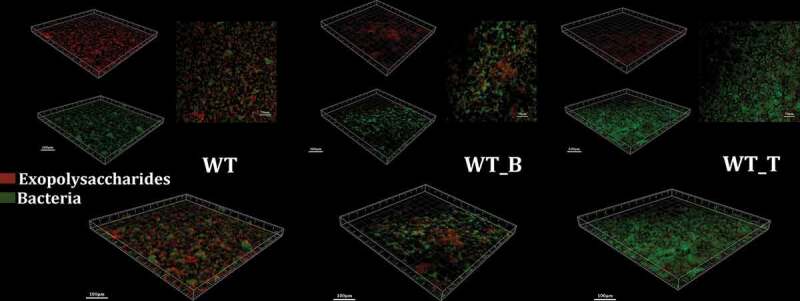
Typical confocal laser scanning microscopy images of 24-hr biofilms of *S. mutans* WT, WT_B and WT_T strain biofilms. All images are three-dimensional projections of bacteria (green) and the glucan matrix (red)

Next, the synthesis and location of exopolysaccharides in mature biofilm of all these three strains were visualized by using a dextran-conjugated cascade dye by CLSM (Figure 4). Since the hypothesis was proposed, that T4 Rnl1 could alter the production and distribution of exopolysaccharides and thus disrupt the biofilm formation of *S. mutans*, 24-h biofilms of WT, WT_B and WT_T were observed. Compared with WT and WT_B strains, the WT_T strain significantly reduced the exopolysaccharides volume and failed to establish a three-dimensional colony skeleton. The overlapping image of exopolysaccharides and bacteria in WT_T was consistent with the result of SEM, presenting a multitude of bare bacteria unwrapped by exopolysaccharides. The results suggested that T4 Rnl1 may play an important role in extracellular polysaccharide assembly.

### The biofilm formation-associated genes are dysregulated at mRNA level in the T4 Rnl1 mutant

**Figure 5.**
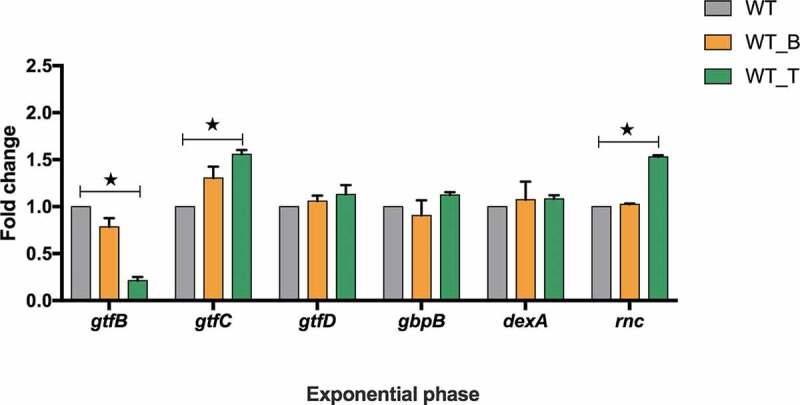
*gtfB/C/D, gbpB, dexA* and *rnc* expression of *S. mutans* WT, WT_B and WT_T strains in the mid-exponential phase measured by qRT-PCR. The results are presented as the mean ± standard deviation. * Kruskal-Wallis test *p*< 0.05

The expression of biofilm formation-associated genes (*gtfB, gtfC, gtfD, gbpB, dexA, rnc*) among all these three strains were quantified and analyzed (Figure 5). Compared with the WT strain, the transcriptional level of *gtfB* in WT_T was significantly decreased, while the transcriptional levels of *gtfC* and *rnc* were increased by 1.7- and 1.6-fold, respectively (Figure 5; *p*< 0.05). The expression levels of other genes in WT_T were similar to those in the WT strain, and the expression levels of all these genes were almost the same as in WT_B and WT (Figure 5; *p*> 0.05).

## Discussion

Overall, our studies suggested that recombinant bacteriophage T4 Rnl1 can effectively impair the biofilm formation of the cariogenic bacterium *S. mutans*. In the present study, the phage therapy was modified by expressing bacteriophage T4 Rnl1 in the host bacterium rather than usage of a specific bacteriophage to attack *S. mutans*. T4 Rnl1 is widely used as a reagent in molecular biology, for its function as a complete 3ʹ-5ʹRNA ligase [9]. Previously, T4 Rnl1 had been successfully expressed in *S. mutans* to detect the potential msRNA-mRNA chimeras [7]. It has been suggested, by comparison with the recombinant bacteriophage T4 Rnl1 in *P. aeruginosa*, that the cell viability of *S. mutans* might be affected. Surprisingly, the 24-h growth curve showed that the impact of T4 Rnl1 on the growth capacity of *S. mutans* was limited (Figure 2a). Consistent with the planktonic growth assay, the cell density within biofilm of WT_T was similar to the WT strain captured by CLSM (Figure 4). The different proliferation ability of carrying vectors, which were used to express T4 Rnl1 in these two strains, might explain why the antimicrobial ability of T4 Rnl1 in *S. mutans* was not as significant as it in *P. aeruginosa.*

Phage therapy is increasingly considered as a viable alternative for the treatment and control of pathogenic bacteria, making therapeutic phage development against oral pathogens possible [5]. Compared with the traditional antimicrobial agents used to attack cariogenic *S. mutans* such as essential oils [20], bioactive fractions [21], and bacteriocins [22], one of the major advantages of phages is that the narrow antibacterial spectrum allows the preservation of the remaining oral microbiome. However, the exquisite specificity of phage therapy against specific pathogens is a major advantage, but also a liability. Up to now, only phages M102, M102AD, e10, f1 and the newly discovered ɸAPCM01 are proven to act against *S. mutans* [3,23,24]. In addition, these phages appear to be serotype-specific, even not all strains of the same serotype are sensitive to a given phage. Moreover, oral phages provide a huge opportunity for the transfer of genetic information into host bacteria, that may lead to the development of antibiotic resistance [25,26]. Thus, the modified phage therapy, expressing bacteriophage virulence traits by transformation and expression of a recombinant vector in host bacteria, may lead to the identification of potential treatment of pathogenic bacteria.

One of the novel aspects of our study is the finding that the recombinant bacteriophage T4 Rnl1 exerts antibiofilm activity instead of bactericidal activity against *S. mutans*. The CV assay detected the decreased the biomass of the 24-h WT_T strain biofilm by comparison with that of the WT strain (Figure 2b). To provide further confirmation, the diminished biofilm structure of the WT_T strain could be seen after 24-h cultivation by SEM (Figure 3). Particularly, the scattered microcolonies of the WT_T biofilm with larger channels surrounded by minor EPS could be seen at high magnification. Indeed, the WT_T biofilm characterized by a dextran-conjugated dye revealed the reduced exopolysaccharides in contrast to the microcolonies enveloped by exopolysaccharides in the biofilms formed by WT after 24-h cultivation (Figure 4). It is suggested that reductions in the exopolysaccharides might have resulted in weakened connections among microcolonies and, therefore, the presence of more scattered microcolonies [27]. Exopolysaccharides produced by *S. mutans* have been recognized as the primary components of the biofilm, for their critical roles in providing mechanical integrity/stability for biofilm formation and a supporting frame for continuous growth of the microcolonies [16,17]. Collectively, it is reasonable to conclude that the bacteriophage T4 Rnl1 decreased the biomass of the biofilms as a consequence of the exopolysaccharide reduction.

The expression of several biofilm-related genes was selected for in-depth investigation. Among the genes that are directed to regulate exopolysaccharides synthesis, the expression of *gtfB* significantly decreased but *gtfC* increased in the WT_T strain. It is confirmed that T4 Rnl1, as the RNA ligase, can link tRNAs to chimeras randomly [6–8]. Thus, we propose that T4 Rnl1 may break down the homeostasis of tRNAs and further dysregulate the mRNAs expression, and the T4 Rnl1-induced tRNAs disorder may account for the inconsistent expression of the *gtf* gene family. It will be very interesting to fully uncover the molecular mechanism of T4 Rnl1-induced genes expression, and our future study will focus on this aspect. As for the *rnc* gene, as a potential regulator of biofilm formation in *S. mutans*, the expression level was significantly up-regulated in the WT_T strain. Consistent with our previous study, the *rnc* gene negatively regulated biofilm formation by posttranscriptional repression of its target genes [19,27].

In summary, the recombinant bacteriophage T4 Rnl1 was identified to be capable of impacting on *S. mutans* biofilms using well-studied biofilm-related factors. To date, anticaries therapies that specifically target biofilm formation of *S. mutans* but do not disturb the cell viability are limited. These data show that the bacteriophage T4 Rnl1 may function as a promising agent for the development of a pathogenic biofilm-specific treatment for dental caries.
